# Assessing the Cost of Helping: The Roles of Body Condition and Oxidative Balance in the Seychelles Warbler (*Acrocephalus sechellensis*)

**DOI:** 10.1371/journal.pone.0026423

**Published:** 2011-10-27

**Authors:** Janske van de Crommenacker, Jan Komdeur, David S. Richardson

**Affiliations:** 1 Behavioural Ecology and Self-Organization, Centre for Ecological and Evolutionary Studies, University of Groningen, Groningen, The Netherlands; 2 Animal Ecology Group, Centre for Ecological and Evolutionary Studies, University of Groningen, Groningen, The Netherlands; 3 Centre for Ecology, Evolution and Conservation, School of Biological Sciences, University of East Anglia, Norwich Research Park, Norwich, England; 4 Nature Seychelles, Mahé, Republic of Seychelles; University of Bristol, United Kingdom

## Abstract

In cooperatively breeding species, helping close relatives may provide important fitness benefits. However, helping can be energetically expensive and may result in increased generation of reactive oxygen species. Consequently, an oxidant/antioxidant imbalance can lead to higher oxidative stress susceptibility. Given the potential costs of helping, it may be that only individuals with a sufficiently good body condition and/or stable oxidative balance can afford to help. Knowledge about relationships between social status and oxidative balance in cooperatively breeding systems is still limited. Studying these relationships is important for understanding the costs of helping and physiological pressures of reproduction. Here we evaluate the relationship between helping behaviour, body condition and oxidative balance in a wild population of the cooperatively breeding Seychelles warbler (*Acrocephalus sechellensis*). In this species, some subordinate individuals help dominant birds with the rearing of young, while others refrain from any assistance. We assessed body condition and oxidative parameters of birds of different social status caught during different breeding stages. We found that, prior to breeding, female subordinates that did not subsequently help (non-helpers) had significantly lower body condition and higher ROMs (reactive oxygen metabolites) than helpers and dominants. During the later stages of breeding, body condition was low in dominants and helpers, but high in non-helpers. Differences in oxidative balance between individuals of different social status were found only during nest care: Dominant males occupied with guarding behaviours tended to have relatively high oxidative stress susceptibility. Furthermore, dominant and helper females showed elevated antioxidant capacity (measured as OXY) in the weeks just prior to egg-laying, possibly representing a change in their reproductive physiology. The results imply that an individuals' oxidative balance may be influenced by factors related to reproduction, which can differ with sex and—within cooperative breeding systems—social status.

## Introduction

In cooperatively breeding species, breeding groups can consist of more than two adult individuals, with subordinate individuals often assisting the dominant breeding pair in the rearing of young [1], [2]. Assistance normally involves provisioning to offspring, but it can also include various other aspects, such as building the nest or defence against predators. Assisting subordinates may be non-reproducing adults (helpers) or reproducing adults that share reproduction with the dominant group members (co-breeders). However, some subordinates within a group (non-helpers) may refrain from helping [3], [4]. The direct [5]–[11] and indirect [12]–[15] fitness benefits of helping and of being helped [1], [9], [16]–[22] are well documented (reviewed in, e.g. [4], [23], [24]).

Although beneficial in many ways, the expression of helping behaviour can be costly in terms of reduced future survival and fecundity [7], [25]–[27] (but see [28]). Physiological costs of helping have also been identified in terms of diminished growth [28]–[30], delayed maturation [1], [31] and hormonal suppression [32]–[34]. More immediately, helping has been shown to be energetically expensive [35]–[39]. Importantly, the energetic costs of helping activities may mean that only individuals that are in sufficiently good condition (i.e., those that can bear the energetic costs / body mass loss without detrimental consequences) can afford to help [28], [38], [40]–[42].

It is hypothesized that levels of energy expenditure/metabolic activity may have considerable consequences for an animal's rate of ageing and life span [43]–[46]. This potential link is thought to be mediated by oxidative stress [47], [48], a mechanism proposed to be a major determinant of life-histories [49]. Greater energetic demands can considerably affect the production of pro-oxidants [49], [50], which are by-products of mitochondrial respiration [51]. Yet, care must be taken before simply using metabolic rate as a proxy for pro-oxidant production, as this link is mediated by other factors such as the respiratory efficiency of the mitochondria [46], [52]. Once formed, pro-oxidants can damage cells and tissues, thereby causing a decline in their ability to function (‘oxidative damage’) [53]–[55], ultimately leading to the onset of degenerative diseases, accelerated senescence and truncated lifespan [46], [48], [56]. Organisms attempt to lower their susceptibility to this continuous oxidative threat by use of an antioxidant defence system; a network of endogenous and exogenous antioxidants that neutralize pro-oxidant radicals [51], [55], [57]. An imbalance between pro-oxidant production and antioxidant defence capacity – and the damage that occurs when the balance favours the former – is referred to as oxidative stress [51], [58].

Substantial variation in oxidative balance may exist between individuals of different social status within breeding groups. Firstly, differences in reproductive behaviour may influence patterns of energy expenditure (e.g., [59]–[62]) and consequently pro-oxidant generation [63] (but see [46], [52]). Secondly, differential energy investment into reproductive behaviour can influence the amount of resources allocated towards self-maintenance (e.g., antioxidant protection) [62], [64], [65]. Moreover, the extent to which individuals can effectively trade-off the allocation of resources (such as energy) towards either reproductive behaviour or self-maintenance is suggested to be closely linked to individual quality or condition [66], which can vary with social status [67], [68]. Pregnancy or egg-production itself may also lead to oxidative imbalance [69]–[72]. To our knowledge the present study is the first to investigate the relationship between social status and oxidative balance in a cooperatively breeding system.

We investigate associations between social status, body condition and oxidative balance in a closed population of Seychelles warblers (*Acrocephalus sechellensis*). On Cousin Island almost every individual has been monitored, colour-ringed and blood sampled since 1997 [73]–[75]. Cooperative breeding occurs on Cousin as a result of a lack of suitable independent breeding vacancies. This drives adult individuals into becoming subordinates within a territory [76]. Within any given breeding season some subordinates help with territory defence and the rearing of young (helpers), while other subordinates refrain from assisting (non-helpers) [14], [15]. Seychelles warblers on Cousin typically produce one clutch per season [73], [77]. Clutches normally consist of one egg (80% of all nests) [73], but on occasion clutches may contain two or three eggs [78]. Molecular analyses have shown that, while subordinate males only very rarely gain paternity, female co-breeding frequently occurs; ca. 44% of subordinate females (co-breeders) gain parentage per year [73]. Behaviour linked to reproduction is expressed by dominants and helpers of both sexes: i.e., nest building and incubation (females only), nest guarding (dominant males) and provisioning the young (both sexes) [79].

We aim to investigate how differences in social status are related to physiological costs over the breeding season, both in terms of body condition and oxidative balance. Specifically we will: (1) test whether pre-nesting body condition and oxidative balance are associated with subordinate helping behaviour over the subsequent breeding season and; (2) investigate the relationship between social status and both body condition and oxidative balance, and examine how these physiological parameters vary across the breeding season. We predict that individual body condition prior to breeding will be related to subsequent helping behaviour in subordinates, as previously shown in meerkats, *Suricata suricatta* [28] and long-tailed tits, *Aegithalos caudatus* [42]. Only individuals that have a sufficiently good initial condition are expected to help. Furthermore, we expect that energy-demanding reproductive activities will result in a lower body condition in all status classes involved in the reproductive attempt (i.e., dominants and helpers, but not non-helpers). Expectations regarding oxidative balance (i.e., the degree of oxidative stress susceptibility) are less clear-cut: over the breeding season, higher workload may lead to higher oxidative imbalance towards pro-oxidants, however, this may not be the case in individuals habituated to high physical activity (e.g., dominants) where mitochondrial down-regulation may occur [46], [52]. In non-helpers, other stressors (e.g., poor nutrition, suppression) could also contribute to oxidant production [67]. Studying these status-related links in a wild population is important as it will provide insights into associations between oxidative balance and helping behaviour as well as reproductive challenges in general.

## Methods

### Ethics Statement

The ethical guidelines promoted by the Association for the Study of Animal Behaviour were followed. The Department of Environment and the Seychelles Bureau of Standards approved all research activities (approval reference A0347).

### Study population and data collection

The study was undertaken on Cousin Island (29 ha; 04°20′ S, 55°40′ E) during the main breeding seasons (July – September) of 2006 – 2009 and the minor breeding periods (January – March) of 2008 and 2009. All 120 territories (containing ca. 320 adult birds) were checked for colour-ringed birds and nesting activity by following the dominant female for a minimum of 15 minutes at least once a week [80]. Active nests were monitored throughout the breeding season to ascertain the breeding stage and social status of all birds present. The dominant male and female were defined as the pair-bonded couple in the territory while the term ‘subordinate’ included all other adult birds resident in the territory [73]. Subordinates were split into two categories: helpers (whether or not co-breeding) and non-helpers [9]. In case of joint nesting, provisioning rates of dominant females and co-breeding helper females are known to be equal, but non-breeding helper females provision significantly less often (23.4% lower feeding rate per hour, *t* = 2.50, *df* = 35, *P*<0.05) [14] and for a shorter period than parents [76]. Average provisioning rates are similar for male and female (non-parent) subordinates [15].

Birds were caught using mistnets. We focused on adult birds (i.e., birds older than 8 months, the minimum age at which birds on Cousin can help or produce young) [81]. Other potentially relevant variables were identified: (i) Territory quality; based on insect prey availability [80], [82], has been shown to be strongly associated with oxidative balance (see [83] for explanation of territory quality calculation); (ii) Sex; (iii) Age (in years): based on the ringing data; (iv) Group size: the number of adults in the territory; (v) Time of day (minutes since sunrise at 6.00 a.m.). For each bird, mass (±0.1 g; using a 50 g Pesola balance), and tarsus length (±0.1 mm; using vernier calipers) were measured. A blood sample (*ca*. 100 µl) was collected immediately after catching by brachial venipuncture. Part of each sample (*ca*. 80 µl) was centrifuged at 8,000 rpm for 8 min. within 3 hours of bleeding, and the plasma obtained was frozen (−18°C) until further analyses. Possible influences of storage time and method on oxidative parameters were tested. Full details regarding the storage and transport of samples are provided in van de Crommenacker *et al.* [83]. The remaining blood was diluted in 1 ml of 100% ethanol in a screw-cap microfuge tube and stored at room temperature. DNA extracted from these samples (following [73]) was used to confirm sex by means of the molecular (PCR) sexing method devised by Griffiths *et al.* [84].

### Analysis of oxidative balance

Oxidative damage and antioxidant capacity were measured using the d-ROMs and OXY-Adsorbent test kits respectively (Diacron, Grosseto, Italy). The most common approach in the measurement of oxidative damage is to quantify the products of free radical reactions with biologic macromolecules [85]. These reactive oxygen metabolites (ROMs) are more stable and thus easier to detect. The d-ROMs test measures the plasma concentration of hydroperoxides, which are a group of ROMs considered to be a marker of lipid and protein oxidative damage [86], [87]. ROMs were analysed by use of the ‘end-point mode’ option in the manufacturer's protocol. The OXY-Adsorbent test quantifies the effectiveness of plasma antioxidants to cope with the oxidant action of hypochlorous acid (HClO). The manufacturer's protocols were followed with a few minor modifications. In brief, 20 µL (ROMs) and 10 µL (OXY) of plasma were used. ROMs are presented as mM of H_2_O_2_ equivalents and OXY as mM HClO neutralized, both calculated from absorbencies measured at 505 nm (spectrophotometer model DU-720, Beckman Coulter). A more detailed description of the protocols can be found in Costantini & Dell'Omo [88]. Inter-assay variation was 2.25% (ROMs) and 1.85% (OXY) and intra-assay variation of 1.68% (ROMs) and 3.08% (OXY). As a few samples were too small to be used in both assays, sample sizes differ slightly between the tests.

### Data analyses

Only individuals that did not switch between territories within the study period were included, which resulted in the exclusion of 23 birds. Some individuals were measured repeatedly on different days (within or across breeding seasons), resulting in a dataset comprising 441 observations (one individual 6 times, 2 individuals 5 times, 4 individuals 4 times, 28 individuals 3 times, 66 individuals twice and 193 individuals measured once). To account for these repeated measurements, a multi-level mixed-modelling procedure (MLWiN 2.20) [89] was used with territory (*n* = 110) and individual identity (*n* = 294) included as random effects. ‘Assay’ (laboratory test session) was not included as a random term, because inter-assay variations were basically similar (ROMs) or lower (OXY) than the intra-assay variations (see preceding methods section). All dependent variables were normally distributed.

To investigate the relationship between social status and condition (Table 1), a model was made with body mass as dependent factor and tarsus length included as a covariate to correct for structural size differences between individuals. This size-corrected body mass is a commonly used indicator of body condition [90] (reviewed in [91]). To investigate the link between social status and the separate oxidative parameters, models were constructed with either ROMs or OXY as the dependent variable (Table 2) and the following explanatory variables: social status (dominant, helper, non-helper), breeding stage (pre-nesting, nest care – from nest-building until hatching –, and provisioning – from hatching until offspring independence –), sex, territory quality, field season (to account for year / season variation), time of day, size-corrected body mass (residual of body mass and tarsus length), group size and age. Territory quality data were log-transformed after which they approximated a normal distribution. Second-order polynomial functions of territory quality, time of day and age were added to the model to allow for quadratic relationships. Model selection was based on the step-wise exclusion of non-significant terms in the order of their significance assessed by their Wald statistic. The final models in Tables 1 and 2 contained the constant and all significant explanatory terms. All eliminated terms were reintroduced to the final model to confirm their lack of contribution. All biologically relevant interactions were tested but only reported when statistically significant (*P*<0.05). Significance levels for post-hoc tests were adjusted for multiple comparisons using the False Discovery Rate (FDR) procedure [92]. In the results section, the adjusted significance level (α_adj_) is stated behind the result in case of hypothesis rejection after FDR adjustment. All post-hoc results are provided in the Supporting Tables S1, S2, and S3.

**Table 1:** Model summary examining associations with body condition in Seychelles warblers.

		Body mass		
	*df*	Estimate ± S.E.	***X*** ^2^	*P*
**Intercept**		**6.22±1.46**		
**Status^1^**	2		**11.19**	**0.004**
Helper		−0.13±0.13		
Non-helper		−0.33±0.10		
**Breeding stage ^2^**	4		**3.99**	**0.14**
Nest care		−0.02±0.10		
Provisioning		−0.21±0.12		
**Sex ^3^**	1	−**0.97±0.14**	**48.35**	**<0.001**
**Season ^4^**	5		**36.62**	**<0.001**
2007 SE season		−0.36±0.11		
2007 NW season		−0.57±0.12		
2008 SE season		−0.16±0.11		
2008 NW season		−0.34±0.12		
2009 SE season		−0.61±0.12		
**Time of day**	1	**0.001±<0.001**	**12.81**	**<0.001**
**Tarsus length**	1	**0.40±0.06**	**51.39**	**<0.001**
Group size	1	−0.02±0.04	0.28	0.60
Age	1	0.02±0.01	1.16	0.28
Territory quality (log)	1	0.13±0.11	1.55	0.21
Age squared	1	−0.001±0.003	0.08	0.77
Territory quality (log) squared	1	0.03±0.15	0.03	0.86
Time of day squared	1	<0.001±<0.001	0.24	0.63
Sex^3^ * Breeding stage^2^	2		**19.12**	**<0.001**
Sex^3^ * Nest care		0.55±0.14		
Sex^3^ * Provisioning		−0.02±0.16		
**Random effects:**				
ó_territory_^2^	1	0.08±0.03	7.90	0.005
ó_individual_^2^	1	0.20±0.05	25.43	<0.001
ó_residual_^2^		0.26±0.03	-	-
^1^ Reference category is ‘dominant’				
^2^ Reference category is ‘pre-nesting’				
^3^ Reference category is ‘male’				
^4^ Reference category is ‘2006 main season’				

Summary derived from a normal response mixed modelling procedure. The final model is shown in bold.

**Table 2:** Model summaries examining associations with oxidative parameters in Seychelles warblers.

		(a) ROMs			(b) OXY		
	*df*	Estimate ± S.E.	***X*** ^2^	*P*	Estimate ± S.E.	***X*** ^2^	*P*
							
**Intercept**		**2.46±0.18**			**123.28±4.11**		
Status^1^	2		5.33	0.07		**0.02**	**0.99**
Helper		0.07±0.09			0.22±5.29		
Non-helper		0.19±0.08			−0.41±3.31		
Breeding stage^2^	2		4.35	0.11		**0.88**	**0.64**
Nest care		0.04±0.06			−2.16±2.92		
Provisioning		−0.10±0.07			−2.84±3.35		
Sex^3^	1	−0.07±0.05	2.24	0.13	**−1.03±2.78**	**0.14**	**0.71**
Territory quality (log)	1	**−0.23±0.07**	**10.07**	**0.002**	−1.55±2.84	0.30	0.59
Season^4^	5		**119.77**	**<0.001**		**28.35**	**<0.001**
2007 SE season		0.06±0.09			8.82±3.34		
2007 NW season		−0.43±0.10			16.24±3.52		
2008 SE season		−0.59±0.09			2.10±3.17		
2008 NW season		−0.37±0.08			4.93±3.24		
2009 SE season		0.01±0.09			5.08±3.39		
Time of day	1	**−0.003±0.001**	**12.98**	**<0.001**	**0.01±0.005**	**6.42**	**0.011**
Time of day squared	1	**<0.001±<0.001**	**19.76**	**<0.001**	−<0.001±<0.001	0.06	0.80
Age	1	**0.05±0.02**	**4.41**	**0.036**	−0.18±0.33	0.30	0.58
Age squared	1	**−0.004±0.002**	**5.37**	**0.021**	0.05±0.08	0.35	0.55
Size-corrected body mass	1	0.05±0.05	1.19	0.28	0.96±1.82	0.28	0.60
Group size	1	0.04±0.02	3.29	0.07	0.96±1.03	0.87	0.35
Status^1^ * Breeding stage^2^	4		7.63	0.11		**10.16**	**0.038**
Helper * Nest care		-			1.81±6.97		
Non-helper * Nest care		-			−12.88±5.02		
Helper * Provisioning		-			0.78±7.39		
Non-helper * Provisioning		-			5.30±6.86		
Sex^3^ * Breeding stage^2^	2		2.31	0.32		**8.61**	**0.014**
Sex^3^ * Nest care		**-**			11.26±3.90		
Sex^3^ * Provisioning		**-**			3.60±4.52		
Territory quality (log) squared	1	0.03±0.11	0.06	0.80	−2.51±4.18	0.36	0.55
**Random effects:**							
ó_territory_^2^	1	0.005±0.01	0.29	0.59	44.84±16.65	12.52	<0.001
ó_individual_^2^	1	0.02±0.02	1.28	0.26	0.00±0.00	-	-
ó_residual_^2^		0.20±0.02	-	-	262.13±20.96	-	-
Reference categories:		^1^ ‘primary’			^3^ ‘male’		
		^2^ ‘pre-nesting stage’			^4^ ‘2006 SE season’		

(a) ROMs (reactive oxygen metabolites) and (b) OXY (antioxidant capacity). Summaries derived from a normal response mixed-modelling procedure. The final models are shown in bold.

Differences in oxidative balance between status groups for each stage of breeding were examined using a three-level structured model, constructed as per the models of Table 2. In the model, ROMs was included as the dependent variable and OXY as a covariate (thus providing an indication of oxidative stress susceptibility (oxidative imbalance), see [93]) along with all explanatory variables left in the final models of Table 2. Furthermore, a bivariate general linear mixed model (GLMM) with both ROMs and OXY included as dependent variables (Table 3) was used to identify covariances across the response variables on the different grouping levels. The model again accounted for all explanatory variables that were left in the final models of ROMs and OXY (from Table 2). Significance of the random terms was tested with likelihood ratio tests. Significance of variances and covariances on the territory and individual level are reported in Table 3. In these likelihood ratio tests, a model with unconstrained covariance was compared with a model where the covariance was constrained to zero.

**Table 3:** Bivariate GLMM examining associations with ROMs and OXY simultaneously in Seychelles warblers.

		ROMs			OXY		
	*df*	Estimate ± S.E.	χ^2^	*P*	Estimate ± S.E.	χ^2^	*P*
**Final model:**							
**Intercept**		2.45±0.18			123.22±4.10		
Status^1^	2		-	-		0.03	0.99
Helper		-			−0.05±5.27		
Non-helper		-			−0.53±3.30		
Breeding stage^2^	2	-	-	-		1.06	0.59
Nest care		-			−2.64±2.91		
Provisioning		-			−2.81±3.34		
Sex^3^	1	-	-	-	−1.13±2.77	0.17	0.69
Territory quality (log)	1	−0.17±0.07	6.16	0.013	-	-	-
Season^4^	5		119.90	<0.001		28.58	<0.001
2007 SE season		0.06±0.09			8.88±3.34		
2007 NW season		−0.43±0.10			16.35±3.52		
2008 SE season		−0.59±0.09			2.14±3.17		
2008 NW season		−0.37±0.08			5.06±3.24		
2009 SE season		0.01±0.09			5.25±3.29		
Time of day	1	−0.003±0.001	13.06	<0.001	0.01±0.005	6.69	0.01
Time of day squared	1	<0.001±<0.001	19.56	<0.001	-	-	-
Age	1	0.05±0.02	4.64	0.031	-	-	-
Age squared	1	−0.004±0.002	5.57	0.018	-	-	-
Status^1^ * Breeding stage^2^	4		-	-		9.43	0.05
Helper * Nest care		-			2.23±6.95		
Non-helper * Nest care		-			−12.65±5.00		
Helper * Provisioning		-			0.64±7.37		
Non-helper * Provisioning		-			4.08±6.84		
Sex^3^ * Breeding stage^2^	2		-	-		9.01	0.011
Sex^3^ * Nest care		-			11.56±3.88		
Sex^3^ * Provisioning		-			4.22±4.51		
**Random effects**							
ó_territory_^2^	1	0.005±0.01	0.26	0.61	44.85±16.65	8.30	0.004
ó_individual_^2^	1	0.02±0.02	2.78	0.10	0.00±0.00	-	-
ó_residual_^2^		0.20±0.02	-	-	262.41±20.98	-	-
**Covariance ROM-OXY**							
cov_territory_	1	0.05±0.27	0.04	0.85			
cov_individual_	1	-	-	-			
cov_residual_		0.67±0.43	2.12	0.15			
**Rejected terms:**							
Status	2		5.70	0.058	-	-	-
Breeding stage	2		4.35	0.11	-	-	-
Territory quality (log)	1	-	-	-	−1.65±2.84	0.34	0.56
Sex^3^	1	−0.07±0.05	2.18	0.14	-	-	-
Age	1	-	-	-	−0.21±0.33	0.41	0.52
Age squared	1	-	-	-	0.05±0.08	0.41	0.52
Time of day squared	1	-	-	-	-<0.001±<0.001	0.08	0.77
Status * Breeding stage	4		7.57	0.11		-	-
Sex * Breeding stage	2		2.40	0.30		-	-
Reference categories:		^1^ ‘primary’			^3^ ‘male’		
		^2^ ‘pre-nesting stage’			^4^ ‘2006 SE season’		

Significant explanatory variables were left in the minimal adequate model after stepwise removal of non-significant variables. Variances and covariances (with standard errors) between the two response variables are given for all random effects.

Finally, results indicated that OXY was elevated in dominant and helper females during nest care. Whether this elevation occurred before or after egg-laying was further investigated using a subset of females for which the exact day of egg-laying was known (*n* = 51). This was not known for all of the nests, because nesting sites are sometimes impossible to reach. In this model all explanatory variables left in the final model of OXY (from Table 2b) were included, except for ‘breeding stage’ which was replaced by ‘pre- versus post egg-laying’. Then a subset of all dominant and helper females that were sampled before egg-laying (*n* = 49) were checked to determine whether there was a relationship between days before egg-laying and OXY. Again all explanatory variables left in the final model of OXY (from Table 2b) were included, except for ‘breeding stage’ that was now replaced by ‘days from egg-laying’.

## Results

**Figure 1:**
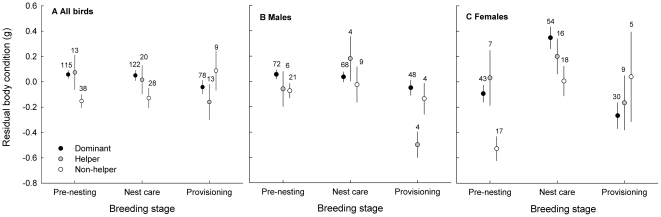
Body condition in relation to social status in Seychelles warblers throughout the breeding season. As a sex-related interaction was found, the figure shows variation in body condition for (a) both sexes, (b) males and (c) females. Dominant birds are indicated in black, helping subordinates in grey and non-helping subordinates in white. To correct for variation caused by factors other than status or breeding stage, residuals from the final model of Table 1 were used. Dots indicate mean ± S.E. and numbers indicate sample sizes.

Status, breeding stage and sex played significant roles in explaining body condition. The three-way interaction between sex, breeding stage and status on body condition was borderline significant (χ^2^_4_ = 9.05, *P = *0.06). Averaged over the entire breeding season, body condition was significantly associated with status (χ^2^_2_ = 11.19, *P* = 0.004) and sex (χ^2^_1_ = 48.35, *P*<0.001), and there was a sex*breeding stage interaction (χ^2^_2_ = 19.12, *P*<0001; Table 1). Post-hoc analyses showed that this interaction resulted from better body condition during nest care in females but not in males (Table S1, Figure 1c). Furthermore, the interaction between status and breeding stage was borderline significant in females only (χ^2^_4_ = 8.96, *P = *0.06; Table S1), indicating that females of different status showed dissimilar patterns of variation in condition over the season (Figure 1c).

The relationship between status and ROMs bordered significance (χ^2^_2_ = 5.33, *P* = 0.07; Table 2a) and there were no interactions between status, breeding stage and sex (all *P*>0.11). Also, there was no relationship between body condition and ROMs (β = 0.05±0.05, χ^2^_1_ = 1.19, *P* = 0.28, Table 2a). Separate analyses per sex (Table S1) revealed that there was a significant association between status on ROMs in females (χ^2^_2_ = 7.00, *P* = 0.03) and a borderline significant interaction between status and breeding stage in males (χ^2^_4_ = 8.52, *P* = 0.07).

**Figure 2:**
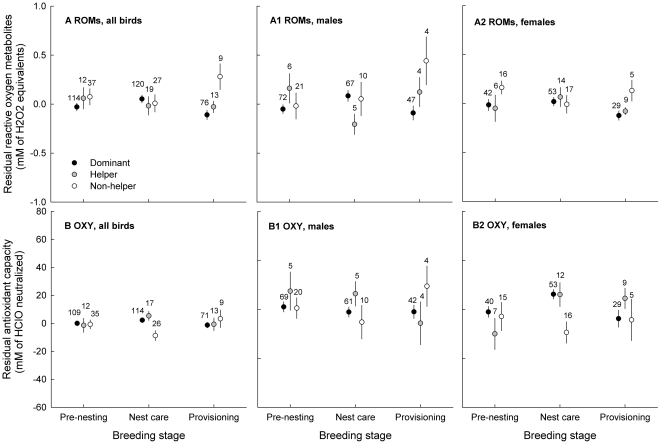
Oxidative parameters in relation to social status in Seychelles warblers throughout the breeding season. Patterns of variation in (a) ROMs (reactive oxygen metabolites) and (b) OXY (antioxidant capacity) are shown. As sex-related interactions were found for OXY, sexes are shown separately. Dominant birds are indicated in black, helping subordinates in grey and non-helping subordinates in white. To correct for variation caused by factors other than status or breeding stage, residuals from the final models of Table 2a and b were used. Dots indicate group mean ± S.E. and numbers indicate sample sizes.

There was no relationship between status and OXY (χ^2^_2_ = 0.02, *P* = 0.99; Table 2b). There were significant interactions between status and breeding stage (χ^2^_4_ = 10.16, *P = *0.038; Table 2b) and sex and breeding stage (χ^2^_2_ = 8.61, *P = *0.014; Table 2b). The three-way interaction between sex, breeding stage and status on OXY was not significant (χ^2^_4_ = 4.97, *P = *0.29). Like ROMs, there was no relationship between body condition and OXY (β = 0.96±1.82, χ^2^_1_ = 0.28, *P* = 0.60, Table 2b). Separate analyses per sex (Table S1) revealed that the sex-related interaction was caused mainly by pronounced elevations in OXY in females but not in males. In females, the interaction between status and breeding stage was (borderline) significant (χ^2^_4_ = 9.54, *P = *0.049), which was likely to result from the increases in OXY during nest care in dominant and helper females, but not in non-helpers (Figure 2b2).

Because of the sex-related interactions for both body condition (Table 1) and OXY (Table 2b), separate plots were made for each sex (Figures 1, 2) and post-hoc analyses were used to further investigate the patterns found.

### Initial (pre-nesting) body condition and oxidative balance

Prior to breeding, female non-helpers had significantly lower body condition than both dominants (*P* = 0.004) and helpers (*P* = 0.002, Figure 1c, Table S2). Pre-nesting female non-helpers also had higher ROMs than pre-nesting female dominants (*P* = 0.011; Figure 2a2). Male non-helpers had a borderline significant tendency to have lower initial body condition than dominants (*P* = 0.045, α_adj_<0.016, Figure 1b). In males no other significant status differences in either initial body condition (all *P*>0.26, Figure 1b) or ROMs (all *P*>0.19, Figure 2a1) were found. There were no pre-nesting status differences in OXY in either sex (all *P*>0.23, Figure 2b).

### Energetic and oxidative patterns over the breeding season

Females had better body condition during nest care compared to the pre-nesting stage (ß = 0.61±0.12, χ^2^_1_ = 24.01, *P*<0.001). Particularly in female non-helpers body condition was lowest prior to breeding, but higher during nest care and provisioning, where it equalled that of female helpers and dominants (Figure 1c, Tables S2, S3). Post-hoc comparisons (see Table S3) between the breeding stages were performed for each status group. Note that when split by sex, the sample sizes for helpers and non-helpers are low, which may complicate the detection of status differences for each breeding stage separately.

#### Dominants

Body condition of dominant males was similar during pre-nesting and nest care, and was slightly higher during provisioning (non-significant once FDR-adjusted: *P* = 0.034, α_adj_<0.016, Figure 1b). ROM levels tended to be lower (again non-significant after FDR correction) during provisioning compared to nest care (*P* = 0.04, α_adj_<0.016, Figure 2a1). No changes in OXY were detected (all *P*>0.43, Figure 2b1). In dominant females, body condition was high during nest care compared to pre-nesting (*P<*0.001, Figure 1c) and then lower during provisioning compared to nest care (*P<*0.001), at a level similar to pre-nesting. There was a non-significant (after FDR adjustment) trend for ROMs to be lower during provisioning than during both nest care (*P* = 0.046, α_adj_<0.033) and pre-nesting stages (*P* = 0.029, α_adj_<0.016, Figure 2a2). OXY followed a similar pattern as body condition: increasing from pre-nesting to nest-care (*P* = 0.028, Figure 2b2) and then reducing from nest care to provisioning (*P* = 0.008) to return to the same level experienced in the pre-nesting stage.

#### Helpers

Male helpers had a significant lower body condition at the end of the breeding season (during provisioning), compared to both pre-nesting (*P* = 0.029) and nest care periods (*P* = 0.003, Figure 1b), however no significant changes in ROMs or OXY were detected (all *P*>0.22, Figure 2a, b). In female helpers (Figure 1c), body condition was equal during pre-nesting and nest care (*P* = 0.78; likely due to low sample sizes, particularly in the pre-nesting stage). Then, during provisioning, body condition was lower again (*P* = 0.014), returning to the same level as pre-nesting. Again, no changes were detected in either ROMs (all *P*>0.23) and OXY (all *P*>0.037, α_adj_<0.016).

#### Non-helpers

In male non-helpers, no changes in either body condition, ROMs or OXY were detected. However, female non-helpers had lower body condition prior to nesting compared to later in the season (nest care (*P* = 0.003)), but no changes in ROMs or OXY were detected.

### Oxidative balance: integrating ROMs and OXY

**Figure 3:**
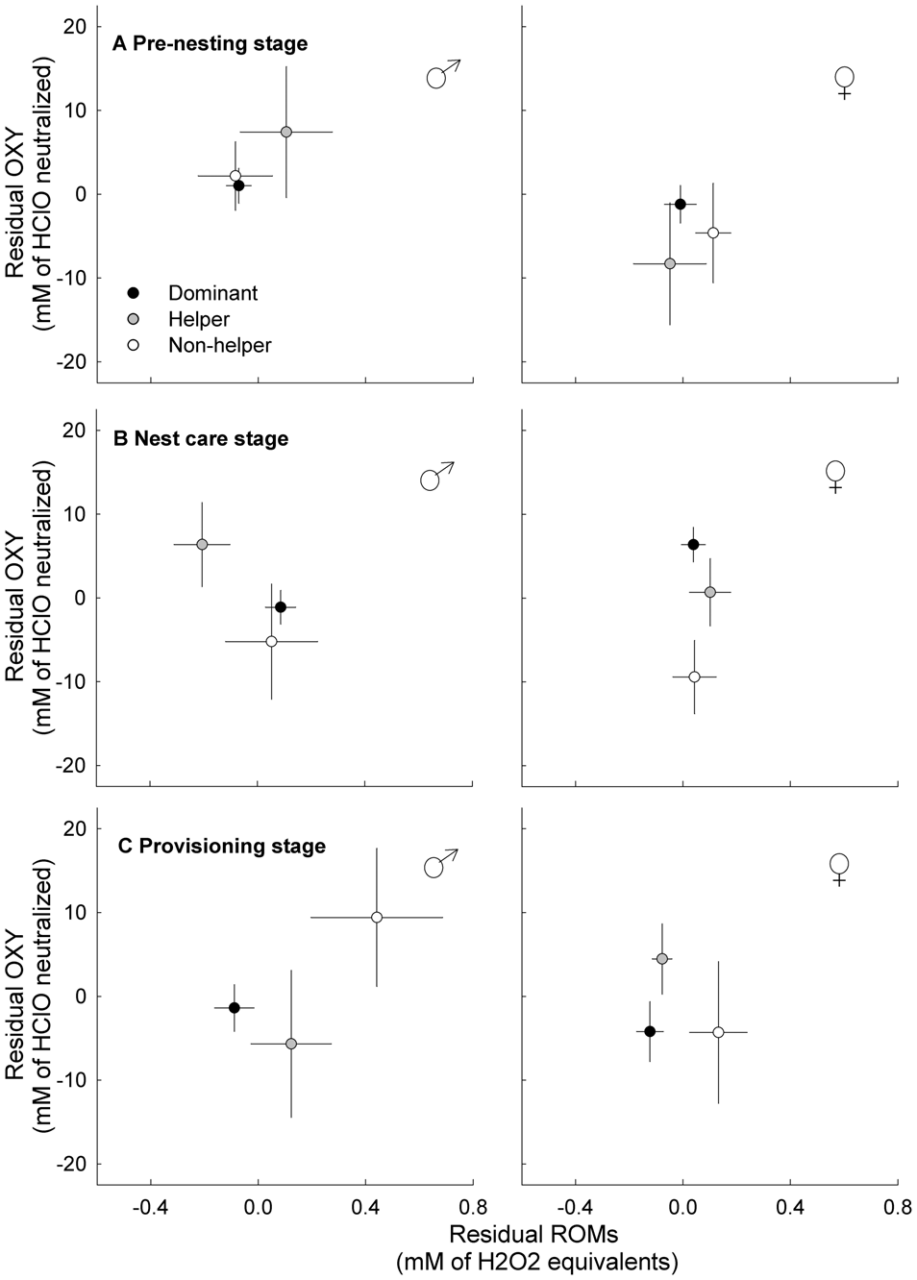
Relationships between ROMs and OXY throughout the breeding season for Seychelles warblers of different status. Patterns of oxidative balance during the (a) pre-nesting stage, (b) nest care stage, and (c) provisioning stage are shown. Sexes are shown separately. Dominant birds are indicated in black, helping subordinates in grey and non-helping subordinates in white. ROMs and OXY are plotted as residuals from the final models of Table 2a and b. Dots indicate group mean ± S.E.

To explore status differences in oxidative balance (i.e., oxidative stress susceptibility), relationships between ROMs and OXY parameters were plotted (Figure 3) per status group and sex over the breeding season. Status differences in oxidative balance were statistically tested using the model in which ROM was the dependent variable and OXY included as a covariate. In the pre-nesting stage, we only found a borderline significant tendency of female non-helpers to have higher oxidative stress susceptibility (oxidative imbalance) than female dominants (*P* = 0.06). The greatest differences in oxidative balance were found during the nest care stage. Female non-helpers had significantly higher oxidative stress susceptibility than female dominants (*P* = 0.011), which was due mainly to the difference in OXY (Figure 3). Male helpers tended to have lower oxidative stress susceptibility than non-helpers (*P* = 0.06) and dominants (*P* = 0.15). During provisioning, there were no significant differences in oxidative balance, either in males (all *P*>0.16) or in females (*P*>0.10) Note again that detection of differences between helpers and non-helpers is difficult due to the low sample sizes. There was no relationship between body condition and oxidative balance (ß = 0.04±0.05, χ^2^_1_ = 0.73, *P* = 0.39).

Testing the relationships of the fixed effects with ROMs and OXY simultaneously in the bivariate GLMM (Table 3) yielded similar results to those in Table 2. Covariances between ROMs and OXY on the random levels were both positive, but non-significant (territory level: between-territory correlation ROM and OXY: *r* = 0.12; ß = 0.05±0.27, *P* = 0.85 and observation level: within-individual correlation ROM and OXY: *r* = 0.09; ß = 0.67±0.43, *P* = 0.15, Table 3). Covariance on the individual level was zero, as a result of the lack of variance in OXY on this level. For both ROMs and OXY, the lowest (observation) level accounted for most of the total variance, but some variance occurred on the territory level (repeatability (or intra-class correlation) ROMs: 0.02, OXY: 0.15), indicating that there were territories in which birds had always higher ROMs than in others. For ROMs, repeatability on the individual level was 0.9, indicating that to a modest degree some individuals always had higher ROMs, possibly reflecting differences in individual quality. For OXY, there was no variance on this level.

### Female antioxidant capacity (OXY) prior to egg-laying

**Figure 4:**
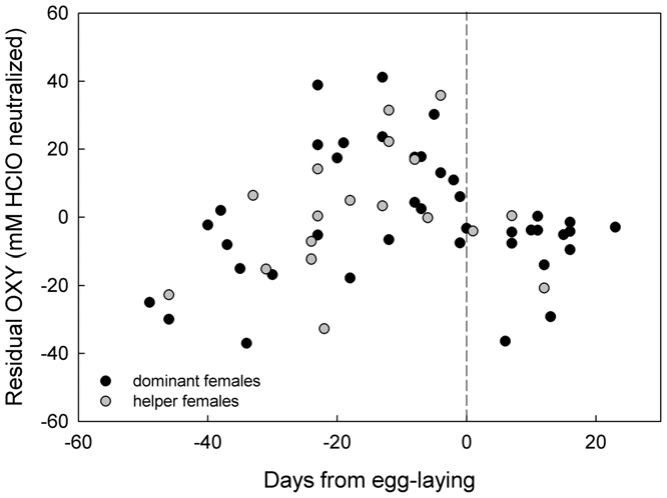
Antioxidant capacity (OXY) in dominant and helper Seychelles warbler females in relation to egg-laying. To correct for variation caused by factors other than status or days from egg-laying, residuals from the final model of Table 2b are used (except for the variables ‘sex’ and ‘breeding stage’). The dashed line indicates the day of egg-laying (day 0).

The timing of the observed elevations in female OXY was explored in more depth with a subset of dominant and helper females for which the exact day of egg-laying was known (*n* = 51). During nest care, pre-egg laying females had significant higher OXY than post-egg laying females (ß = 20.59±4.43, n_pre-egg_ = 32, n_post-egg_ = 19, χ^2^_1_ = 21.58, *P*<0.001) and there was no difference between dominants and helpers (χ^2^_1_ = 0.01, *P* = 0.94). In another subset of dominant and helper females sampled before egg-laying (*n* = 49, both pre-nesting and nest care stage), there was a positive correlation between days before egg-laying and OXY: the closer to egg-laying the higher OXY (ß = 0.94±0.20, χ^2^_1_ = 21.92, *P*<0.001). This correlation did not differ between dominant and helpers (χ^2^_1_ = 3.89, *P* = 0.47). Plotting OXY against the days from egg-laying (Figure 4) showed that the elevation in OXY mainly manifested itself from ca. 25 days before egg-laying until the egg was laid.

### Other factors associated with body condition and oxidative parameters

Body condition and oxidative parameters were also associated with factors other than social status, sex and breeding stage (Tables 1, 2). Birds had better body condition (Table 1) and OXY (Table 2b) later in the day. ‘Time of day’ was also significantly associated with ROMs, but here the relationship was quadratic and ROMs were highest early and late in the day (Table 2a). There was a negative linear relationship between territory quality and ROMs: ROMs were higher in lower territory quality conditions (Table 2a). There was a negative quadratic relationship between age and ROMs, with ROMs being low in young individuals increasing in middle-aged birds and decreasing again in old individuals (Table 2a). Furthermore, all dependent factors were significantly associated with season (Table 1, 2).

Possible influences of storage time and method on oxidative parameters were found to be non-significant (time period between bleeding and centrifuging; ROMs: *P* = 0.48, OXY: *P* = 0.33; cooling method during this time period (i.e., fridge versus cool box); ROMs: *P* = 0.76, OXY: *P* = 0.89; time period between sample collection and assaying; ROMs: *P* = 0.96, OXY: *P* = 0.30). Quadratic relationships were all non-significant (all *P*>0.27).

## Discussion

### Pre-nesting body condition and oxidative balance

Our study on the Seychelles warbler indicates a relationship between the expression of helping behaviour and body condition prior to breeding. Female subordinates that did not help in the subsequent season had significantly lower pre-nesting body condition than female subordinates that did. This may indicate that only subordinate females with a sufficiently good body condition can afford to help. As helping behaviour is energetically costly [35]–[39], levels of helping behaviour are likely to be condition-dependent [28], [38], [94]. The decision over whether to help is thought to be based on a trade-off between investing available energy into selfish behaviour (including self-maintenance) or into cooperation [95]. Individual condition is assumed to be an important proximate factor influencing this, as has been shown in other correlational studies where individuals in better condition were more likely to help [3], [42]. Furthermore, experiments increasing condition through supplementary feeding increased the probability that an individual would help [40], [41].

We found no such status differences in the pre-nesting body condition of males. This difference could be explained by the fact that female investment in helping in this species may be higher than for males, particularly in terms of nest building and incubation [78]. The costs of helping are therefore assumed to be higher for females, thus explaining why condition prior to breeding would be a more important determinant of helping for females than for males. Helping could also be more costly for females because they may be doing more than just assisting the dominant pair. It may be that when female subordinates are in a sufficiently good condition they also attempt to co-breed (lay an egg in the nest), a reasonably common occurrence in this species [96]. Such co-breeding female subordinates will also suffer from the cost of direct reproduction. Moreover, these co-breeding subordinates will always invest in helping throughout the whole breeding season, irrespective of their own egg's fate [9], [73] (as early egg or chick loss is frequent in multi-egg nests in this species [97]). The potential for female subordinate direct fitness though co-breeding could also link female condition and helping: subordinate females with sufficiently good body condition may afford not only to help but also to produce an egg. Unfortunately we are currently unable to rule out this possibility. Even when parentage data on nestlings (sampled at day 8–14) is available to detect co-breeding [73], significant egg loss and / or early brood reduction means that cases where the subordinate female has laid an egg – and therefore perceives herself to be a co-breeder – will be missed.

Interestingly, non-helping females, but not males, had higher ROMs in the pre-nesting stage than both dominants (significant) and helpers (borderline significant). Due to their higher ROMs (and no elevation in OXY), non-helpers tended to have higher oxidative stress susceptibility than female dominants prior to nesting (see Figure 3), which may be another important motive for subordinates not to help. Viewed from a physiological trade-off perspective, energetically-intensive reproductive behaviours are thought to exert pressure on the maintenance of antioxidant defences [62], [64], [65], [71] (reviewed in [72]). Therefore investment into self-maintenance (i.e. protection against oxidative damage) may gain priority over investment into helping. Indeed, body condition in non-helping females was better later in the breeding season, perhaps as a result of this selfish investment.

### Energetic and oxidative patterns linked to helping over the breeding season

#### Body condition

Status, breeding stage and sex all played significant roles in explaining body condition. Body condition was, on average over the breeding season, best in dominants, then helpers and poorest in non-helpers. This finding matched our expectations and the general pattern found in literature [34], [98]–[100]. We found no correlation between body condition and any of the oxidative parameters, which is perhaps not surprising. One might hypothesize that birds in better condition would have lower oxidative stress susceptibility, but heavier birds may also have to bear the oxidative consequences of carrying more weight while flying. Hence, the relationship between body condition and oxidative balance might not be straightforward.

Helpers and dominants of either sex had lower body condition during the provisioning stage compared to the earlier breeding stages. That body condition was lowest during provisioning, when energy constraints are commonly assumed to be greatest [101]–[103], is perhaps not surprising. However, although body condition losses are normally considered as evidence of physical workload, there is also a possibility that birds deliberately reduce their body mass in an adaptive response to diminish the flight costs involved in feeding young [104], [105]. However, such intentional strategy can still be considered as a cost, which can only be endured by individuals that are in sufficiently good condition and have the ability to effectively acquire resources when needed (e.g., for their own energetic demands) [105].

Males and females showed different patterns of body condition variation throughout the breeding season, and status differences in these patterns were more pronounced in females than in males (i.e., the interaction between status and breeding stage was borderline significant in females but not in males; Table S1). Interestingly, helper and dominant females (but not non-helpers) increased in body condition during the nest care stage, but again had a lower body condition during provisioning. This may reflect that breeding in this species is timed to coincide with the peak insect prey availability [79]. Higher food intake should allow females to better cope with the metabolic requirements of egg production [106]–[108]. Another explanation for the observed increase in mass would be gonadal growth and/or egg production [109], [110] (reviewed in [104]).

#### ROMs

Averaged over the breeding season, the overall relationship between status and ROMs bordered significance (Table 2a) and there were no interactions of status, breeding stage and sex. However, separate analyses per sex revealed that status differences in ROMs throughout the breeding season were most obvious in males, as was shown by the borderline significant status*breeding stage interaction. ROMs in dominant males were high during nest care, whereas helpers had significantly lower ROMs. This resulted in a tendency for oxidative stress susceptibility to be the lowest in male helpers, and higher in non-helpers and dominants (Figure 3). Given the correlational nature of the present study we can only speculate about the cause of these status-related variations in oxidative balance. Possibly, higher workload of dominants may have resulted in higher production of ROMs. In the nest care period dominant males are occupied with guarding their fertile mate and nest, while helpers do not mate guard and are less involved in nest defence [111]. This guarding behaviour in dominants is likely to be energetically intensive and to be traded off with time allocation into foraging behaviour [97]. The acute stress that comes with warding off competitors/predators, and the associated hormonal elevations (e.g., testosterone [112], [113]) may further enhance ROM generation [114]. However, the ‘higher workload, higher ROMs’ concept is not necessarily the right explanation as shown during the provisioning stage. Here, the dominant males – that are known to contribute more to provisioning than subordinates – had the lowest ROMs, whereas non-helpers had the highest ROMs. One may argue that provisioning may be less energy-intensive than expected, but it is also possible that the energy spent during provisioning is not directly translated into high ROMs. The relationship between metabolism and oxidant production is complex and depends on habituation of the organism to elevated metabolism [49]. Where acute workload in unaccustomed individuals can lead to higher oxidant production [50], [115], those used to high activity (i.e., dominants) may diminish oxidative effects through, for example, mitochondrial down-regulation or enhancement of antioxidant defences ([52], [116]–[118]; reviewed in [119]). In Seychelles warblers, this may mean that the higher investments in reproduction and territory defence in dominants does not result in higher ROMs as would be the case in non-habituated individuals.

The highest ROM levels during provisioning were found in non-helpers (both sexes) – individuals free from any feeding duties. This same trend was also found over the entire breeding season; ROMs lowest in dominants, followed by helpers and highest in non-helpers, a pattern most pronounced in females. This result is also contrary to the hypothesis of ROMs being positively related to reproductive investment. The high ROMs in non-helpers could be interpreted in different ways. Poorer nutrition of non-helping subordinates could contribute to pro-oxidant augmentation, for example through oxidant release in metabolic organs [120], [121] or psychological stress of food insecurity [67], [122]. However, although non-helpers did have the highest ROM levels during provisioning, their body condition during this period was not lower than dominants and helpers. Possibly, other stressors may be important [123]. For example, in contrast to helpers that may help to ‘pay’ the dominants to stay within the social group [5], non-helpers (that by definition do not pay this ‘rent’) may be marginalized or forced out of the territory [124], [125]. However, this seems unlikely as aggression by dominants towards non-helping subordinates in the Seychelles warbler is extremely rare (D. Richardson & J. Komdeur, unpublished data). The differences in ROMs could also be due to status differences in hormones, which are potential mediators of oxidant production [114], [126]–[128]. Further research may reveal the possible role of hormones (e.g., corticosterone, testosterone) in the mediation of ROMs production; however, experimental manipulations would be unfeasible in this endangered species.

#### OXY

Antioxidant capacity was expected to respond to the pro-oxidants produced, but in reality ROMs and OXY did not follow equal patterns. Interactions with OXY were found between status and breeding stage and between sex and breeding stage. The most patent patterns of OXY were exhibited by females (i.e., the interaction status*breeding stage interaction was significant in females but not in males). Female dominants and helpers showed markedly higher antioxidant levels during nest care compared to female non-helpers (Figure 2b2), which resulted in lower oxidative stress susceptibility (Figure 3). Post-hoc investigations in these nest-caring dominant and helper females showed that OXY was elevated only prior to egg-laying (Figure 4), which may represent changes in the female's reproductive physiology. The production of eggs requires deposition of yolk lipids [129]. These lipids and associated proteins, which are transferred through the blood to the ovarian follicles, can also serve as antioxidants [129] and this could have led to the increase in plasma OXY values measured. In birds, females are also known to allocate large amounts of antioxidants into their eggs [130], [131], and the female's oxidative condition has been shown to correlate with the antioxidant content of the egg [132]–[134]. This may be an important maternal investment to enhance the antioxidant machinery of the offspring [130], [135]. However, it is unclear to what extent our measured values of OXY are influenced by such active antioxidant up-regulation. The high OXY levels were not necessarily associated with high ROM levels or *vice versa*, as was shown by the non-significant covariation on the random levels of the bivariate GLMM (Table 3). This again shows that relationships between pro- and antioxidants can differ greatly over the season, which may depend on their source and utility. When antioxidant increases are related to egg-production, their correlation with ROMs may be different (i.e., independent) than when they are being synthesized solely as a response to ROMs in order to ensure oxidative protection. This implies that reproductive status is an important factor to take into consideration during the evaluation of oxidative parameters in animals.

To conclude, in the cooperative breeding Seychelles warbler we found that pre-nesting body condition was better in female subordinates that helped in the subsequent breeding season than in those that did not. Then, in the individuals that opted not to help, body condition was better towards the end of the breeding season, eventually being equal (or better) than all status classes that were reproductively active (including subordinate helpers). We also detected other status differences in condition over the breeding season, which may reflect the cost of expressing reproductive behaviours. Patterns of oxidative parameters were less straightforward. We found interesting relationships implying that regulation of the complex oxidative balance may be influenced by factors related to reproduction. These results concur with the general patterns and theories established through studies of oxidative stress in more tractable systems and indicate that similar patterns and principles occur in wild living organisms.

To our knowledge this study is the first to examine relationships between social status and oxidative parameters in a natural population of a cooperative breeder. It is important to note that the exploration presented here is based on correlational data, as is typical for studies in natural settings. The data provide an indication of patterns of oxidative balance variation in relation to social status, but the results emphasize the complexity of the oxidative stress framework, making it extremely difficult to draw conclusions about causation and the adaptive nature of the responses. Further experimental work is now needed to disentangle the causes and effects of links between measures of condition and the expression of helping behaviour in this and other species.

## Supporting Information

Table S1 **Investigation of associations between social status, breeding stage and physiological indices for each sex separately.** If non-significant, the status*breeding interaction was eliminated to investigate the main effects of status and breeding stage. The models further included all explanatory variables were left in the final models of Tables 1 and 2.
(DOC)

Table S2 **Post-hoc tests: status differences in physiological indices per breeding stage and for each sex.** Significance levels were adjusted for multiple comparisons using the False Discovery Rate (FDR) procedure.
(DOC)

Table S3 **Post-hoc tests: patterns throughout the breeding season per status group and for each sex.** Significance levels were adjusted for multiple comparisons using the False Discovery Rate (FDR) procedure.
(DOC)
