# Supplementary material for: Assessing the Cost of Helping: The Roles of Body Condition and Oxidative Balance in the Seychelles Warbler (Acrocephalus sechellensis)
Source: PLoS One. 2011 Oct 27;6(10):e26423. doi: 10.1371/journal.pone.0026423 (PMC3203150; doi:10.1371/journal.pone.0026423)
Supplement: Table S1 — Investigation of associations between social status, breeding stage and physiological indices for each sex separately. If non-significant, the status*breeding interaction was eliminated to investigate the main effects of status and breeding stage. The models further included all explanatory variables were left in the final models of Tables 1 and 2. (DOC) [file pone.0026423.s001.doc]

**Table S1.**

|  |  |  | **Body condition** | | |  | **ROMs** | | |  | **OXY** | | |
| --- | --- | --- | --- | --- | --- | --- | --- | --- | --- | --- | --- | --- | --- |
|  |  | *df* | Estimate ± S.E. | *χ*^2^ | *P* |  | Estimate ± S.E. | *χ*^2^ | *P* |  | Estimate ± S.E. | *χ*^2^ | *P* |
|  |  |  |  |  |  |  |  |  |  |  |  |  |  |
| Males | **Status^1^** | 2 |  | **7.42** | **0.025** |  |  | 1.22 | 0.54 |  |  | 0.61 | 0.74 |
|  | Helper |  | -0.37 ± 0.18 |  |  |  | 0.08 ± 0.14 |  |  |  | 3.81 ± 5.01 |  |  |
|  | Non-helper |  | -0.28 ± 0.13 |  |  |  | 0.13 ± 0.12 |  |  |  | -0.36 ± 3.44 |  |  |
|  | **Breeding stage^2^** | 2 |  | **7.93** | **0.019** |  |  | 2.59 | 0.27 |  |  | 1.08 | 0.58 |
|  | Nest care |  | -0.03 ± 0.09 |  |  |  | 0.12 ± 0.08 |  |  |  | -2.92 ± 2.94 |  |  |
|  | Provisioning |  | -0.27 ± 0.10 |  |  |  | 0.01 ± 0.09 |  |  |  | -2.48 ± 3.50 |  |  |
|  | **Status^1^ * Breeding stage^2^** | 4 |  | 6.45 | 0.17 |  |  | 8.52 | 0.07 |  |  | 3.75 | 0.44 |
|  | Helper * Nest care |  | 0.33 ± 0.40 |  |  |  | -0.56 ± 0.31 |  |  |  | 0.95 ± 11.66 |  |  |
|  | Non-helper * Nest care |  | 0.11 ± 0.24 |  |  |  | -0.07 ± 0.20 |  |  |  | -4.50 ± 7.59 |  |  |
|  | Helper * Provisioning |  | -0.65 ± 0.41 |  |  |  | 0.02 ± 0.33 |  |  |  | -12.57 ± 12.45 |  |  |
|  | Non-helper * Provisioning |  | 0.11 ± 0.34 |  |  |  | 0.56 ± 0.28 |  |  |  | 11.11 ± 10.29 |  |  |
|  |  |  |  |  |  |  |  |  |  |  |  |  |  |
|  |  |  |  |  |  |  |  |  |  |  |  |  |  |
| Females | **Status^1^** | 2 |  | **10.08** | **0.006** |  |  | **7.00** | **0.030** |  |  | 1.44 | 0.49 |
|  | Helper |  | 0.04 ± 0.15 |  |  |  | -0.13 ± 0.13 |  |  |  | -8.34 ± 6.97 |  |  |
|  | Non-helper |  | -0.40 ± 0.14 |  |  |  | -0.33 ± 0.10 |  |  |  | -1.66 ± 5.07 |  |  |
|  | **Breeding stage^2^** | 2 |  | **37.75** | **<0.001** |  |  | 4.41 | 0.11 |  |  | **9.19** | **0.010** |
|  | Nest care |  | 0.58 ± 0.12 |  |  |  | -0.02 ± 0.10 |  |  |  | 8.02 ± 3.64 |  |  |
|  | Provisioning |  | -0.16 ± 0.16 |  |  |  | -0.21 ± 0.12 |  |  |  | -2.63 ± 4.51 |  |  |
|  | **Status^1^ * Breeding stage^2^** | 4 |  | 8.96 | 0.06 |  |  | 4.56 | 0.34 |  |  | **9.54** | **0.049** |
|  | Helper * Nest care |  | -0.54 ± 0.36 |  |  |  | 0.13 ± 0.23 |  |  |  | 8.50 ± 8.83 |  |  |
|  | Non-helper * Nest care |  | 0.10 ± 0.29 |  |  |  | -0.25 ± 0.18 |  |  |  | -15.55 ± 7.11 |  |  |
|  | Helper * Provisioning |  | -0.34 ± 0.39 |  |  |  | 0.13 ± 0.25 |  |  |  | 16.27 ± 9.47 |  |  |
|  | Non-helper * Provisioning |  | 0.94 ± 0.39 |  |  |  | 0.19 ± 0.25 |  |  |  | -0.20 ± 9.60 |  |  |
|  |  |  |  |  |  |  |  |  |  |  |  |  |  |
|  |  |  |  |  |  |  |  |  |  |  |  |  |  |
| ^1^ Reference category is 'dominant' | | | | | | | | | | | | | |
| ^2^ Reference category is 'pre-nesting' | | | | | | | | | | | | | |
