# Supplementary material for: Assessing the Cost of Helping: The Roles of Body Condition and Oxidative Balance in the Seychelles Warbler (Acrocephalus sechellensis)
Source: PLoS One. 2011 Oct 27;6(10):e26423. doi: 10.1371/journal.pone.0026423 (PMC3203150; doi:10.1371/journal.pone.0026423)
Supplement: Table S2 — Post-hoc tests: status differences in physiological indices per breeding stage and for each sex. Significance levels were adjusted for multiple comparisons using the False Discovery Rate (FDR) procedure. (DOC) [file pone.0026423.s002.doc]

**Table S2.**

|  |  |  | **Pre-nesting** | |  | **Nest care** | |  | **Provisioning** | |
| --- | --- | --- | --- | --- | --- | --- | --- | --- | --- | --- |
|  |  |  | *χ*^2^_1_ | *P* |  | *χ*^2^_1_ | *P* |  | *χ*^2^_1_ | *P* |
| **Body mass** | Both sexes | Dominants* - Helpers | 0.005 | 0.94 |  | 0.44 | 0.51 |  | 1.90 | 0.17 |
|  |  | Dominants* - Non-helpers | **9.55** | **0.002** |  | **6.34** | **0.012** |  | 0.30 | 0.59 |
|  |  | Helpers* - Non-helpers | 3.12 | 0.08 |  | 1.81 | 0.18 |  | 2.00 | 0.16 |
|  |  |  |  |  |  |  |  |  |  |  |
|  | Males | Dominants* - Helpers | 1.29 | 0.26 |  | 0.01 | 0.91 |  | **8.75** | **0.003** |
|  |  | Dominants* - Non-helpers | 4.00 | 0.05 |  | 0.83 | 0.36 |  | 0.40 | 0.53 |
|  |  | Helpers* - Non-helpers | 0.001 | 0.98 |  | 0.47 | 0.49 |  | 3.12 | 0.08 |
|  |  |  |  |  |  |  |  |  |  |  |
|  | Females | Dominants* - Helpers | 1.95 | 0.16 |  | 0.39 | 0.53 |  | 0.09 | 0.76 |
|  |  | Dominants* - Non-helpers | **8.21** | **0.004** |  | **5.43** | **0.020** |  | 1.17 | 0.28 |
|  |  | Helpers* - Non-helpers | **9.51** | **0.002** |  | 1.98 | 0.16 |  | 0.54 | 0.46 |
|  |  |  |  |  |  |  |  |  |  |  |
| **ROMs** | Both sexes | Dominants* - Helpers | 1.03 | 0.31 |  | 0.1 | 0.75 |  | 1.06 | 0.30 |
|  |  | Dominants* - Non-helpers | 3.78 | 0.05 |  | 0.15 | 0.70 |  | **9.62** | **0.002** |
|  |  | Helpers* - Non-helpers | 0.09 | 0.77 |  | 0.32 | 0.57 |  | **3.71** | **0.05** |
|  |  |  |  |  |  |  |  |  |  |  |
|  | Males | Dominants* - Helpers | 1.72 | 0.19 |  | 1.46 | 0.23 |  | 1.28 | 0.26 |
|  |  | Dominants* - Non-helpers | 0.46 | 0.50 |  | 0.02 | 0.88 |  | **5.98** | **0.014** |
|  |  | Helpers* - Non-helpers | 0.66 | 0.42 |  | 1.27 | 0.26 |  | 1.06 | 0.30 |
|  |  |  |  |  |  |  |  |  |  |  |
|  | Females | Dominants* - Helpers | 0.002 | 0.96 |  | 0.96 | 0.33 |  | 0.64 | 0.42 |
|  |  | Dominants* - Non-helpers | **6.51** | **0.011** |  | 0.72 | 0.40 |  | **6.17** | **0.013** |
|  |  | Helpers* - Non-helpers | 3.04 | 0.08 |  | 0.002 | 0.96 |  | 2.92 | 0.09 |
|  |  |  |  |  |  |  |  |  |  |  |
| **OXY** | Both sexes | Dominants* - Helpers | 0.002 | 0.96 |  | 0.20 | 0.66 |  | 0.04 | 0.85 |
|  |  | Dominants* - Non-helpers | 0.02 | 0.90 |  | **12.22** | **<0.001** |  | 0.66 | 0.42 |
|  |  | Helpers* - Non-helpers | 0.01 | 0.91 |  | **8.12** | **0.004** |  | 0.28 | 0.60 |
|  |  |  |  |  |  |  |  |  |  |  |
|  | Males | Dominants* - Helpers | 0.68 | 0.41 |  | 0.88 | 0.35 |  | 0.38 | 0.54 |
|  |  | Dominants* - Non-helpers | 0.002 | 0.96 |  | 0.58 | 0.45 |  | 1.39 | 0.24 |
|  |  | Helpers* - Non-helpers | 0.62 | 0.43 |  | 1.63 | 0.20 |  | 1.75 | 0.19 |
|  |  |  |  |  |  |  |  |  |  |  |
|  | Females | Dominants* - Helpers | 1.43 | 0.23 |  | 0.001 | 0.98 |  | 1.55 | 0.21 |
|  |  | Dominants* - Non-helpers | 0.11 | 0.74 |  | **12.34** | **<0.001** |  | 0.05 | 0.82 |
|  |  | Helpers* - Non-helpers | 0.74 | 0.39 |  | **7.35** | **0.007** |  | 1.07 | 0.30 |
|  |  |  |  |  |  |  |  |  |  |  |
| * = Reference category for post-hoc comparison | | | | | | | | | | |
