# Supplementary material for: Assessing the Cost of Helping: The Roles of Body Condition and Oxidative Balance in the Seychelles Warbler (Acrocephalus sechellensis)
Source: PLoS One. 2011 Oct 27;6(10):e26423. doi: 10.1371/journal.pone.0026423 (PMC3203150; doi:10.1371/journal.pone.0026423)
Supplement: Table S3 — Post-hoc tests: patterns throughout the breeding season per status group and for each sex. Significance levels were adjusted for multiple comparisons using the False Discovery Rate (FDR) procedure. (DOC) [file pone.0026423.s003.doc]

**Table S3.**

|  |  |  | **Dominants** | |  | **Helpers** | |  | **Non-helpers** | |
| --- | --- | --- | --- | --- | --- | --- | --- | --- | --- | --- |
|  |  |  | *χ*^2^_1_ | *P* |  | *χ*^2^_1_ | *P* |  | *χ*^2^_1_ | *P* |
|  |  |  |  |  |  |  |  |  |  |  |
| **Body mass** | Both sexes | Pre-nesting* and Nest care | **8.37** | **0.004** |  | 0.41 | 0.52 |  | 2.11 | 0.15 |
|  |  | Pre-nesting* and Provisioning | **5.84** | **0.016** |  | 3.88 | 0.049 |  | 1.22 | 0.27 |
|  |  | Nest care* and Provisioning | **27.17** | **<0.001** |  | **8.64** | **0.003** |  | 0.01 | 0.93 |
|  |  |  |  |  |  |  |  |  |  |  |
|  | Males | Pre-nesting* and Nest care | 0.28 | 0.60 |  | 0.53 | 0.47 |  | 0.07 | 0.79 |
|  |  | Pre-nesting* and Provisioning | 4.46 | 0.034 |  | **4.74** | **0.029** |  | 0.12 | 0.73 |
|  |  | Nest care* and Provisioning | 3.19 | 0.07 |  | **9.14** | **0.003** |  | 0.24 | 0.62 |
|  |  |  |  |  |  |  |  |  |  |  |
|  | Females | Pre-nesting* and Nest care | **16.91** | **<0.001** |  | 0.08 | 0.78 |  | **9.06** | **0.003** |
|  |  | Pre-nesting* and Provisioning | 1.92 | 0.17 |  | 2.70 | 0.10 |  | 3.65 | 0.055 |
|  |  | Nest care* and Provisioning | **28.05** | **<0.001** |  | **5.96** | **0.014** |  | 0.02 | 0.88 |
|  |  |  |  |  |  |  |  |  |  |  |
| **ROMs** | Both sexes | Pre-nesting* and Nest care | 1.33 | 0.25 |  | 0.44 | 0.51 |  | 0.44 | 0.51 |
|  |  | Pre-nesting* and Provisioning | 2.83 | 0.09 |  | 0.43 | 0.51 |  | 1.56 | 0.21 |
|  |  | Nest care* and Provisioning | **7.87** | **0.005** |  | 0.003 | 0.96 |  | 2.72 | 0.10 |
|  |  |  |  |  |  |  |  |  |  |  |
|  | Males | Pre-nesting* and Nest care | 3.05 | 0.08 |  | 1.85 | 0.17 |  | 0.18 | 0.67 |
|  |  | Pre-nesting* and Provisioning | 0.21 | 0.65 |  | 0.01 | 0.93 |  | 3.61 | 0.06 |
|  |  | Nest care* and Provisioning | 4.06 | 0.04 |  | 1.35 | 0.25 |  | 2.19 | 0.14 |
|  |  |  |  |  |  |  |  |  |  |  |
|  | Females | Pre-nesting* and Nest care | 0.001 | 0.98 |  | 0.36 | 0.55 |  | 2.61 | 0.11 |
|  |  | Pre-nesting* and Provisioning | 3.99 | 0.046 |  | 0.19 | 0.66 |  | 0.04 | 0.84 |
|  |  | Nest care* and Provisioning | 4.79 | 0.029 |  | 1.42 | 0.23 |  | 0.85 | 0.36 |
|  |  |  |  |  |  |  |  |  |  |  |
| **OXY** | Both sexes | Pre-nesting* and Nest care | 2.01 | 0.16 |  | 0.67 | 0.41 |  | 4.41 | 0.036 |
|  |  | Pre-nesting* and Provisioning | 0.13 | 0.72 |  | 0.001 | 0.98 |  | 0.44 | 0.51 |
|  |  | Nest care* and Provisioning | 2.71 | 0.10 |  | 0.76 | 0.38 |  | 4.3 | 0.038 |
|  |  |  |  |  |  |  |  |  |  |  |
|  | Males | Pre-nesting* and Nest care | 0.62 | 0.43 |  | 0.02 | 0.89 |  | 0.99 | 0.32 |
|  |  | Pre-nesting* and Provisioning | 0.39 | 0.53 |  | 1.52 | 0.22 |  | 0.78 | 0.38 |
|  |  | Nest care* and Provisioning | 0.003 | 0.96 |  | 1.27 | 0.26 |  | 2.23 | 0.14 |
|  |  |  |  |  |  |  |  |  |  |  |
|  | Females | Pre-nesting* and Nest care | **4.85** | **0.028** |  | 4.34 | 0.037 |  | 1.54 | 0.22 |
|  |  | Pre-nesting* and Provisioning | 0.34 | 0.56 |  | 2.50 | 0.11 |  | 0.11 | 0.75 |
|  |  | Nest care* and Provisioning | **7.08** | **0.008** |  | 0.14 | 0.70 |  | 0.30 | 0.59 |
|  |  |  |  |  |  |  |  |  |  |  |
| * = Reference category for post-hoc comparison | | | | | | | | | | |
